# Drug Repositioning: New Approaches and Future Prospects for Life-Debilitating Diseases and the COVID-19 Pandemic Outbreak

**DOI:** 10.3390/v12091058

**Published:** 2020-09-22

**Authors:** Zheng Yao Low, Isra Ahmad Farouk, Sunil Kumar Lal

**Affiliations:** 1 School of Science, Monash University, Bandar Sunway, Subang Jaya 47500, Selangor Darul Ehsan, Malaysia; zylow1@student.monash.edu (Z.Y.L.); iaf1@student.monash.edu (I.A.F.); 2 Tropical Medicine & Biology Platform, Monash University, Subang Jaya 47500, Selangor Darul Ehsan, Malaysia

**Keywords:** drug repositioning, novel diseases, orphan diseases, drug personalisation, COVID-19, HIV, Cushing’s syndrome

## Abstract

Traditionally, drug discovery utilises a de novo design approach, which requires high cost and many years of drug development before it reaches the market. Novel drug development does not always account for orphan diseases, which have low demand and hence low-profit margins for drug developers. Recently, drug repositioning has gained recognition as an alternative approach that explores new avenues for pre-existing commercially approved or rejected drugs to treat diseases aside from the intended ones. Drug repositioning results in lower overall developmental expenses and risk assessments, as the efficacy and safety of the original drug have already been well accessed and approved by regulatory authorities. The greatest advantage of drug repositioning is that it breathes new life into the novel, rare, orphan, and resistant diseases, such as Cushing’s syndrome, HIV infection, and pandemic outbreaks such as COVID-19. Repositioning existing drugs such as Hydroxychloroquine, Remdesivir, Ivermectin and Baricitinib shows good potential for COVID-19 treatment. This can crucially aid in resolving outbreaks in urgent times of need. This review discusses the past success in drug repositioning, the current technological advancement in the field, drug repositioning for personalised medicine and the ongoing research on newly emerging drugs under consideration for the COVID-19 treatment.

## 1. Introduction

Drug development is a lengthy and detailed process that focuses on treating diseases by creating specific molecules that target the disease to alleviate or treat the presenting symptoms. Drug discovery is simply denoted as a process whereby an active ingredient is identified, primarily by high throughput screening, resulting in a biomolecule that targets a particular cellular pathway to treat a particular disease [1,2]. The process is designed to ensure that a new drug is effective, safe and most importantly available for patient use in the shortest possible time. In the act of drug discovery, de novo design has always been the foundation of study to generate novel and constructive bioactive molecules from the ground up. This approach involves stimulating the 3D structure of a receptor against which the drug is designed initially with the aid of computer modelling techniques followed by extensive research on the chemistry or molecular biology/biochemical formulation. Drug design involves the uncovering of the structure of target complexes and modifications of lead compounds via computational aid. Indisputably, with the vast improvement in technology and now techniques offered such as recombinant DNA, genomics and combinatorial chemistry, drug discovery has advanced in precision and has significantly decreased the time and overall cost of new drug development [3].

Nonetheless, this conventional de novo approach is still a stumbling block to many big pharmaceutical and biotechnology companies as it is an utterly time-consuming, laborious, high-risk and costly process. For instance, it usually costs an average of US$2.6 billion for approximately 17 years of development from molecule to market. Even so, only 2.01% of all drug development initiatives finally make it to the market as a successful drug [4]. To add to this, the resource-intensive process requires high public demand to make it worth the effort as well as pressure on the industry to produce a drug that is affordable and significantly profitable for the drug producer. Research and development costs have always been a critical success factor for a new drug. Unfortunately, the number of new drugs approved per dollar invested has decreased significantly since the mid-1990s despite the enormous spending and investment in research and development [5].

The above factors have driven numerous drug developers to pursue different alternative approaches for new drug discoveries. One such example is improvising existing drugs with an enhanced formulation such as extended-release and better dosing frequency. The existing drug Valproate IR (immediate-release) has been modified to ER (extended-release) for the treatment of epilepsy. Valproate ER has driven numerous promising outcomes with drug compliance of up to 71% as compared to 40% on the IR version [6]. Another example is the drug Concerta (extended-release methylphenidate) for hyperactive disorder. It has been significantly improved in dosing frequency over the traditional methylphenidate like Ritalin, which requires up to three doses daily as opposed to a single daily dose for Concerta [7]. Also, being an extended-release drug, Concerta significantly improves drug adherence of patients and reduces side-effects such as nausea, vomiting, high blood pressure, irritability, sweating and numbness as it is gradually released in the blood. 

Most recently, a new approach called drug repositioning has been gaining popularity [5]. Drug repositioning (DRS), otherwise also known as drug repurposing, drug recycling, drug redirecting, drug re-tasking or drug reprofiling, is currently denoted as an alternate approach to find new uses of an original drug to treat other diseases aside from the intended ones [8,9]. This new idea has gravitated towards a trend, especially for old and established drugs, to seek new disease targets. The positive projection of drug repositioning lies within the effort of drug developers to fulfil demands, especially for diseases that lack drug treatment or are otherwise expensive to treat. Of course, profit is an inseparable factor here. As the Nobel laureate pharmacologist James Black once said: “The most fruitful basis for the discovery of a new drug is to start with an old drug” [10]. This sudden spurt of interest is evident from PubMed, where there are more than 1000 publications as of the year 2020 on “drug repositioning”. For instance, the drug Sildenafil, more commonly known as Viagra, is a potent example of a repositioned drug; it was first intended to treat heart disease and can now be used to treat erectile dysfunction. Another prime example is Duloxetine, currently the most preferred drug therapy for stress urinary incontinence (SUI), where it was initially developed for depression [5]. 

Drug repositioning plays a very crucial role in drug development, as it lowers the overall developmental costs by approximately $300 million. Also, drug repositioning results in lower development risk as the safety of the repositioned drugs has already been well established in humans and other preclinical models. Apart from that, this approach has a much shorter development timeline, as there is no need to repeat the safety assessment and formulation development protocols since they are already in place [11]. Furthermore, drug repositioning dictates high investment potential on old drugs, which were initially forfeited especially for long-term safety reasons. For instance, the Thalidomide tragedy which became widespread across Europe, Australia and Japan reported more than 10,000 children with an inborn defect known as phocomelia, where the limbs were severely underdeveloped or absent [12]. This drug was subsequently banned in 1962. However, with a safer and more careful approach, the company Celgene successfully repositioned Thalidomide into an effective cancer drug therapy for multiple myeloma. In the year 2003 alone, it successfully captured $271 million in revenue [13].

Apart from the obvious, drug repositioning offers a positive outcome in orphan drug development. Orphan diseases (ODs) are those that affect only a minute population, for which no effective drug treatments are available. There are several factors that contribute to this scenario. As such, the low drug demand from the rare occurrence add up to prohibitive costs in drug development, and low subject availability for clinical trials, subsequently driving interest away from the researchers and pharmaceutical industries as the investment of time and money may not be profitable. With drug repositioning, the ever-concerning cost and time issue for an orphan drug can be resolved by exploring new uses from the existing or abandoned drug therapies. 

One of the most significant examples is the currently ongoing global outbreak of the novel coronavirus disease, COVID-19. In current studies, commercially available drugs used to treat other diseases such as HIV and malaria are being carefully evaluated as treatments for COVID-19. Multiple types of drugs such as antimalarials, antivirals and antibiotics are being considered as potential treatments for COVID-19 either individually or in combination. Many are undergoing clinical trials to be evaluated for safety and efficacy in humans. An early example in research was the use of Hydroxychloroquine (HCQ) in combination with azithromycin, which generated deep interest in the potential of Chloroquine (CQ) and its derivates for consideration as a potential treatment for COVID-19 [14]. There are many advantages to adopting similar drug repositioning tactics for improved healthcare globally. In this review, we discuss how drug repositioning plays a crucial role in tackling epidemics and pandemics where little or no treatment is available, for orphan diseases and personalised medicine. Also, we present the current state of research, significance, prospects and challenges in drug repositioning.

## 2. Technological Advancements of Drug Repositioning—Current Methods in Research

**Figure 1:**
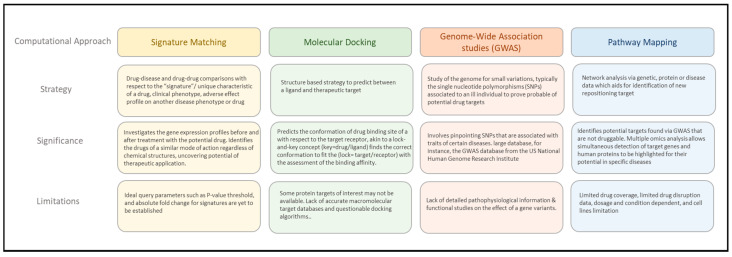
A summary of the strategy, significance and limitations of common computational approaches in drug repositioning.

Concerning the advancements in cell-based screening, multiplex assays, data mining, in-silico bioinformatics and cheminformatics databases, pharmaceutical industries have shown an increased interest in exhuming compounds that were once failed in development due to many reasons [15]. Typically, there are three steps before considering repositioning a drug: (1) the identification of candidate molecules for the given indication; (2) the theoretical assessment of the drug effect in preclinical models; and (3) the evaluation of safety efficacy in phase II clinical trials [11]. The approach of drug repositioning can be subdivided into two main categories, the computational and experimental approaches. Using these approaches individually or in combination, it can form hypotheses on generating candidates for drug repositioning. A computational approach is typically driven by a massive amount of data, which entails the use of the genetic expression, chemical structure and proteomic data of the drug candidates. There are a few computational approaches that are widely used today, such as signature matching, computational molecular docking, genome-wide associated studies (GWAS), pathway mapping, retrospective clinical analysis and novel sources of drug repurposing studies [11]. On the contrary, the experimental approaches that are widely used today are binding assays and phenotypic screenings [11]. In this review, we focus on a few computational approaches (shown in Figure 1) that add value to drug repositioning.

### 2.1. The Computational Approach to Drug Repositioning

#### 2.1.1. Signature Matching

Signature matching is a technique that utilises transcriptomic, proteomic/metabolomic data and chemical profiles to make drug–disease and drug–drug comparisons with respect to the “signature” or unique characteristic of the drug, disease or clinical phenotype [16]. The transcriptomic signature can be utilised for a drug–disease and drug–drug comparison. Firstly, the drug–disease comparison investigates the gene expression profiles before and after treatment with the potential drug. Subsequently, result comparisons are made with the disease-associated expression profile against the healthy expression profile. The hypothesis is that if a set of gene expression changes within a disease, matches with a drug with the opposite set of gene expression changes, i.e., genes upregulated in disease are downregulated by the drug, that renders potential in that drug to be used therapeutically for the disease [17]. This method has successfully identified and exploited many drug repositioning opportunities, especially on anticancer drug-resistant signatures [11]. This approach mainly relies on the signature reversion principle (SRP), in which a drug capable of reversing the hallmark of a gene expression pattern for a particular disease phenotype is then very likely to be able to revert the given disease phenotype. A study conducted by Wei et al. (2006) showed that the drug profile of an mTOR inhibitor, Rapamycin, matched the signature of glucocorticoid sensitive profile and successfully reversed the glucocorticoid resistance in acute lymphoblastic leukaemia cells when screened across the drug-associated gene expression database. The screening result implied that Rapamycin might be a promising treatment for lymphoid malignancies [18]. On the other hand, the drug–drug comparison strategy identifies drugs with a similar mode of action regardless of the variation in chemical structures, in a quest to uncover the potential of a drug in therapeutic application [19]. This approach renders a vast opportunity in drug repositioning and, of course, relies heavily on accessible gene expression databases such as the National Institutes of Health (NIH) Library of Integrated Network-based Cellular Signatures [11]. Apart from that, the chemical profiles are also extremely important for drug repositioning, in which chemical structures with respect to the biological activities of two different drugs are compared. This process is carried out to understand the chemical similarities and subsequently construct a network based on the shared chemical features to predict new targets for the established drugs [16]. The aforementioned approaches have significantly benefited the selection of potential candidates for drug repositioning. Nevertheless, a notable limitation of this approach is the difficulty in determining the optimal query signature, in which the parameters can vary remarkably between different diseases and studies. Ideal query parameters such as the *p*-value threshold and absolute fold change for signatures are yet to be established. For instance, a central nervous system injury involves 21 genes for signature, based on a *p*-value threshold of 0.05 and an absolute fold change of ≥1.5. In contrast, glioblastoma, an aggressive cancer in the spinal cord or brain can engage up to 1000 genes with a *p*-value threshold of 0.0001 and an absolute fold change of ≥4 [20]. 

#### 2.1.2. Molecular Docking

As the word “docking” suggests, molecular docking involves a computational strategy in predicting the conformation on the binding site of a drug to the target receptor. Molecular docking is akin to a lock-and-key concept where the key (drug/ligand) finds the correct orientation to fit the lock (target/receptor). Molecular docking involves the prediction of the ligand conformation with reference to its position and orientation within the target sites along with the assessment of the binding affinity [21]. This approach helps to identify novel interactions that might be potential candidates for drug repositioning. As such, a study conducted by Dakshanamurthy et al. (2012) discovered that the anti-inflammatory agent, Celecoxib, binds to cadherin-11, a crucial adhesion molecule in rheumatoid arthritis and poor prognosis malignancies, upon performing high-throughput computational docking on 3671 FDA-approved drugs across 2335 human protein crystal structures. This strategy enhances the drug repositioning opportunity, especially for diseases with no existing therapies [22]. More recently, with the emergence and spread of COVID-19, molecular docking has successfully determined a potential antiviral for COVID-19, a poly-ADP-ribose polymerase-1, CVL218, an inhibitor that binds to the N-terminal of the nucleocapsid (N) protein of the virus [23].

However, challenges may arise when some protein targets of interest might not be available or there is a lack of accurate macromolecular target databases or questionable docking algorithms. Therefore, it is advised to perform more than one computational approach when screening for drug targets rather than relying on molecular docking alone. 

#### 2.1.3. Genome-Wide Association Studies (GWAS)

Genome-wide association study (GWAS) is a study of the genome for small variations, typically the single nucleotide polymorphisms (SNPs) of an ill individual. GWAS involves the examination across the genome-wide set of genetic variants in different individuals to pinpoint any variations that are associated with a trait of certain diseases [24]. The construction of GWAS genes associated with the disease traits involves an extensive database [24]. For instance, the GWAS database from the U.S. National Human Genome Research Institute was used in the study conducted by Sanseau and colleagues [24]. The database studied by Sanseau et al. includes an extensive collection of publications accompanied by >4800 rows of data with respect to a trait and SNP [24]. Upon investigation, they were 991 GWAS genes identified relevant to the disease traits. The analysed rows of GWAS data that corresponded to the trait and SNPs are subsequently investigated for the druggable potential or otherwise. Then, 15.6% of the 991 selected GWAS genes are associated with drug projects in pharmaceutical pipelines, in which 51 GWAS traits are different from the drug indication for 92 genes, giving opportunities to drug repositioning [24]. For example, the drug Denosumab, which was initially intended for postmenopausal osteoporosis, is now a potential drug candidate for Crohn’s disease associated with variations in tumour necrosis factor member 11 (*TNFSF11*), discovered with the aid of GWAS [23]. In another study, a similar approach was used with three different drug-target databases (DrugBank, Therapeutic Target Database and PharmGKB) and a gene prediction platform known as *Gentrepid*. The study successfully discovered 184/192 predicted candidate genes as novel therapeutic targets for coronary artery disease (CAD), and 981/993 potential drugs from the drug databases for repositioning, giving new directions in repositioning existing drugs for CAD at a reasonable cost [25]. Nonetheless, GWAS has its limitations where detailed pathophysiological information is absent, and the lack of functional studies on the effect of a gene variant. Hence, GWAS data should be used in concert with other computational approaches in predicting targets for drug repositioning [11]. 

#### 2.1.4. Pathway Mapping

Pathway mapping has been frequently used with GWAS to identify potential drugs. In some cases, the identified potential targets found via GWAS are not druggable. Pathway mapping is a network analysis on drug or disease networks based on gene expression patterns, disease pathology and protein interaction, providing the necessary information in exploiting drug repositioning opportunities. Pathway mapping provides vital information for genes that are either upstream or downstream of the GWAS-associated target, subsequently unravelling the potential for drug repositioning [24]. A study on respiratory viral infections conducted by Smith et al. successfully identified 67 common biological pathways that may be associated with respiratory viral infections. Subsequently, crossing these pathways to the DrugBank database, few potential drug repositioning candidates are discovered for the treatment of respiratory viral infection. One of them is the drug Amrinone, a phosphodiesterase inhibitor that was used for the treatment of congestive heart failure [26]. Also, multiple omics analysis mapped to significant pathways allows for the simultaneous detection of target genes and human proteins for their potential in specific diseases. For instance, drug candidates and human protein targets have been identified for several diseases such as dengue haemorrhagic fever [27], head and neck squamous cell carcinoma [28] and rheumatoid arthritis [29] amongst many others, using this approach. However, there are also downsides to this approach. Limitations include drug coverage, drug disruption data, dosage, condition-dependent and cell line limitations as animal model expression patterns may not reflect the human systems [30]. 

### 2.2. Potential Computational Construct to Drug Repositioning

Varieties of computational methods for drug repositioning have been discovered and proposed over the years with the aim of improving efficiency, reducing limitations and fulfilling the duty of drug repurposing. Recently, a novel computational method which utilises machine learning-based methods, matrix decomposition-based methods and network-based methods accompanied by various algorithms for drug repositioning was developed, known as tensor decomposition [31]. This method was proposed by Wang and colleagues in 2019, and it has been outperforming several baseline algorithms such as SNScore, Network-based Random Walk with Restart on Heterogeneous network (NRWRH) and Collective Matrix Factorization (CMF) in recovering missing associations. Also, it solves the difficulty in training negative samples in deep learning methods such as the multi-layer perceptron, deep belief network and stacked auto-encoder for drug repositioning in recent years. It involves the construction and decomposition of three-dimensional tensors which entails the associations among the trios, drugs, targets and diseases (DTD) [31]. Latent factors followed by topological data analysis (TDA) are then taken into account to cluster and investigate the properties of drugs, targets and diseases in their respective functional groups. This approach is able to recover not only the missing associations of drugs, target and diseases but can also predict new drug repositioning candidate and discover adverse drug effects, primarily on cancers [31]. For instance, it was found that Leflunomide (LEF), a drug for active rheumatoid arthritis, might increase the risk for pancreatic neoplasms upon investigating the latent factors and compared to KEGG NETWORK drug database followed by validation through several studies. Furthermore, when there is a small latent factors outcome, it can adhere to the fundamental assumption in drug repositioning where similar drugs and targets have similar functional effects. Repositioning can be done on drugs to diseases that come from two clusters with strong associations, instead of assuming new drug–disease associations only. To define clusters, diseases with a similar molecular mechanism tends to appear in the same cluster [31]. This effort was made by finding drugs with a similar chemical structure to the disease-associated drugs. This approach provides a vast potential and new insight to drug repositioning [31]. 

Apart from computational approaches, experimental approaches are a supplement in drug repositioning. A study conducted by Niu and colleagues has generated a favourable outcome by combining both approaches on drug repositioning for hyperuricemia [32]. Hyperuricemia is a disorder where excessive uric acid levels are accumulated in the blood, as a result of abnormal purine levels or a failure of normal uric acid excretion. The key enzyme that regulates uric acid production is the xanthine oxidase (XOD) which catalyses the conversion of hypoxanthine to xanthine, and subsequently uric acid. A protein-ligand docking software by Idock was used in this study to screen approved drugs structures attained from the ZINC database, DSSTox database and the NCGC Pharmaceutical Collection (NPC) [32]. A total of 3167 drugs approved for clinical use were sorted for best-scoring compounds with two criteria, a low Idock score with respect to binding free energy and a high RF score with respect to binding affinity [32]. Subsequently, the potential drug candidate underwent a series of in vitro and in vivo assays carried out in different concentrations on hyperuricemic mice [32]. The study concluded that Olsalazine Sodium (OS), a drug intended for acute and chronic ulcerative colitis has massive potential in inhibiting XOD, providing a potential hypouricemic agent for future application [32].

### 2.3. Artificial Intelligence in Drug Repositioning

With the advancement of Computational Artificial Intelligence, the barrier and limitation of a computational approach for drug repositioning can also be vastly reduced. Artificial intelligence (AI) can be simply defined as the mimicking of human intelligence demonstrated by machines which entail reasoning, planning, learning and perception to maximise the chances of achieving different goals under extreme load and urgencies like pandemics or diseases of unknown aetiology. The emergence of AI is inevitable, especially in the context of the ever-complex process of drug discovery and repositioning. With the ever-expanding large pool of chemical and biological data, the need for powerful computing and algorithms for data mining is urgent to ensure a fast, high efficiency and low-cost drug discovery process [33].

While AI is a part of computer-aided drug design (CADD), this computational approach has been applied for drug discovery and repositioning for decades. The prime machine learning (ML) theories, such as the logistic regression (LR), naive Bayesian classifier (NB), k-nearest neighbors (KNN) algorithm, multiple linear regression (MLR), Gaussian process [23] and many more have relied on AI integration for the purposeful interpretation of drug repositioning opportunities [34,35]. Today, the ML theory, accompanied by the advancement of AI, have moved forward to deep learning (DL) methods which promote stronger data processing accompanied by reliable results in the shortest possible time and with high cost-efficiency. For instance, with the use of the deep neural network (DNN), Ma and colleagues have successfully overcome a few limitations. As such, the difficulty in training and screening a large number of compounds accompanied by the improvement in the prediction accuracy of the quantitative structure-activity relationship (QSAR) method is essential for predicting on- and off-target activities in drug discoveries [36]. With that being said, drug–target interaction (DTI) identification is a critical aspect to look at in drug repositioning. The DTI in drug repositioning lies within the traditional ligand and structure-based methods which utilise the QSAR to predict biological activities of target molecules with the assumption of structurally similar molecules that give almost similar biological activities and authentic molecular docking imitations [33]. However, the limitations in these traditional computational methods draw attention to the DL methods. For instance, a DL based algorithm framework namely, the DeepDTIs, has successfully predicted new DTIs between approved drugs and targets accurately without the need to separate the targets into different classes. The DeepDTIs has also been proven to outperform the other ML methods such as the random forest [37] and support vector machine (SVM). This approach provides a vast potential to be further used for new drug targets prediction based on existing targets, or new target interaction with existing drugs [38].

In urgent times of need, like the current COVID-19 pandemic outbreak, AI has been a huge supplement to drug repositioning. For instance, BenevolentAI, an AI prediction method based on an extensive repository of structured medical database which includes various connections extracted by scientific literature via machine learning, has aided in the search of potential drug repositioning candidates for the treatment of COVID-19 [39]. The study has shown a vital key for the SARS-CoV-2 infection, known as the ACE-2 cell receptor, found abundantly in the blood vessels, heart and lung AT2 alveolar epithelial cells, subsequently leading to COVID-19 [39]. The AP2-associated protein kinase 1 (AAK1), regulates the ACE-2 cell receptor, a crucial receptor for the inhibition of SARS-CoV-2 entry [40]. A Janus kinase inhibitor, namely Baricitinib was discovered via this approach, which suggests good therapeutic dosing at 2–4 mg once daily, to inhibit the AAK1, contributing to an effective treatment with low unpleasant side-effects [39,40]. In sum, the computational approaches accompanied by AI show great promise for drug repositioning, especially amid the COVID-19 outbreak with an urgent need for a therapeutic proposition. Also, in some cases, this approach is comparable or even better than the in vitro assays.

## 3. Advantages of Drug Repositioning

Drug repositioning has shown many benefits for various diseases throughout the years since it emerged. Apart from being beneficial for patients that suffer from the lack of drug treatments, drug repositioning is also beneficial to the economy in many ways. In this section, we will discuss these benefits and provide examples of repositioned drugs from the past for various reasons including serendipitous discoveries, orphan diseases and the current drugs in repositioning development for the new COVID-19 disease. 

### 3.1. Cost and Time Saving

**Figure 2:**
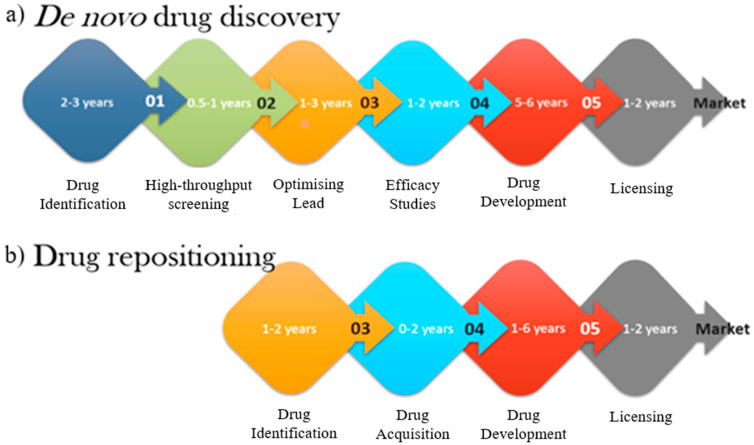
(**a**) Traditional de novo drug discovery takes up to 17 years from drug identification to market. (**b**) In-vitro and in-vivo screening, validation, lead optimisation, and efficacy studies require significantly lesser time thus resulting in significant time overall time saved and reduced overall cost.

The current costly and time-consuming paradigm of drug discovery has restricted the larger outcome of drug development. With the substantial costs, high risk and slow pace in drug discovery, an alternative approach is much needed to revive drug development to fulfil market needs [41]. Additionally, the desperately low number of approved new molecular entities that remain almost stagnant, coming from 26 new drugs approved from 1976 to only 27 new drugs approved in 2013, puts drug repositioning at an advantage [42]. This condition further exacerbates as the number of new drugs approved per billion USD deteriorate significantly to below 1 after the year of 2000, according to Eroom’s Law, indicating higher spending on research and development with significant less new molecular entities approval per year [43]. Moreover, up to $20 billion annual sales were reported in 2012 for the repositioning of failed drugs by various pharmaceutical companies [8]. In sum, drug repositioning confers to considerable improvement in development times and cost accompanied by maximising revenue and reduced risk (Figure 2). 

As shown in Figure 2, drug repositioning shortens the time and cost needed at many stages as compared to the traditional de novo approach. This remarkable outcome is a result of the repositioned candidates that have gone through rigorous safety and pharmacokinetic profile studies for clinical development and have been well established but never made it through due to unfortunate failures at the endpoint on the intended target [44]. This new development course of repositioning can circumvent the unexpected derailment due to unforeseen failures during primary preclinical studies [45]. With the established formulations, ADMET (absorption, distribution, metabolism, excretion, toxicity) data and the omission of preclinical/phase I of drug development, drug repositioning significantly reduces the time needed and subsequently saves drug discovery costs up to 40% from molecule to market via a shorter and faster pathway [3,10].

### 3.2. Accessible Bioinformatics Databases for Drug Candidates

There are vast opportunities that arise from drug repositioning, in which researchers can pinpoint existing drugs that recognise specific targets based on the prior discoveries, without having to perform major experiments. This is achievable with the ever-expanding bioinformatics and cheminformatics databases such as the proteomic database (UniProt), genomic databases (Entrez-Gene) and pharmaceutical databases (DrugBank/Drug Central/PubChem), providing the necessary gene expression and chemical structures, in which relevant candidates can be screened for drug repositioning [46]. For instance, with the aid of these databases, tricyclic antidepressants were identified to treat renal carcinomas aside from small-cell lung cancers (SCLC) [47]. These unconventional in-silico strategies for drug repositioning are driven by massive amounts of data generated via various data mining tools and web technologies, such as PubMed Boolean-type online searching tools, making the retrieval of information more accessible [48].

## 4. Classic Examples of Repositioned Drugs

A notable success in drug repositioning can be dated back to as early as the 1970s, where the drug Minoxidil was approved to treat hair loss aside from its intended use for the treatment of hypertension. The success of drug repositioning continued in the 1990s and 2000s with the release of Sildenafil, a repositioned drug to treat erectile dysfunction aside from the initial indication, angina followed by the drug, Duloxetine, used for stress urinary incontinence which was originally intended for major depressive disorder. 

### 4.1. Minoxidil, Farewell to Hair Loss

The classic example of success in drug repositioning is Minoxidil, an oral drug that was intended to treat hypertension then became a treatment for hair loss prevention [49,50]. Minoxidil was first introduced in the 1970s/1980s to counteract hypertension. However, patients who had consumed Minoxidil came across a widespread side effect known as hypertrichosis, which caused hair growth on the face, arms, shoulders and legs in both men and women [51]. The active ingredient that contributed to this encounter was minoxidil-sulphate, which caused the potassium channels to open [51]. This serendipitous encounter on hair growth, especially on balding men, was latterly repositioned and approved by the FDA in 1979 as a topical application for hair loss (androgenic alopecia) treatment [13,52,53]. Its mechanism functioned by causing vasodilation via the opening of potassium channels, which indirectly upregulated vascular endothelial growth factor (VEGF) expression in human hair follicle dermal papilla cells (HFDPC) of the human scalp [54], thereby promoting significant hair growth. 

### 4.2. Sildenafil, New Usage for Erectile Dysfunction

Another prominent repositioned drug is Sildenafil, a phosphodiesterase type 5 inhibitor developed by Pfizer, which was initially intended to treat angina, a condition characterised by chest pain and discomfort due to underlying heart disease where one or more coronary arteries are blocked or narrowed. Sildenafil works by relaxing the coronary arteries to improve coronary blood flow. However, it opened up possibilities for the treatment of erectile dysfunction when several volunteers experienced a strong, unusual persistent erection upon Sildenafil consumption [55]. With further studies and trials carried out, Sildenafil had whopping annual sales of $1.88 billion in the U.S. alone. Today, Sildenafil is widely available over the counter. It works primarily as a treatment for erectile dysfunction via the same mode of action by inhibiting phosphodiesterase type 5 AND preventing the breakdown of cyclic guanosine monophosphate (cGMP), a blood flow regulator in the penis. As of 2019, according to GoodRx, Pfizer’s Viagra still manages to capture a staggering 65% market share even after its loss in exclusive rights on the drug in 2017. 

### 4.3. Duloxetine Repositioned for Stress Urinary Incontinence

Duloxetine was a norepinephrine and serotonin reuptake inhibitor developed by Eli Lilly & Company and marketed as Cymbalta in August 2004. Its prime intention was to treat major depressive disorder [56]. However, serotonin and norepinephrine are also known to exhibit excitatory effects on urethral sphincter motor neurons, thereby increasing urethral resistance. With a series of preclinical studies conducted by Eli Lilly, the aforementioned hypothesis came to a fruitful outcome. The drug can now be used to treat stress urinary incontinence (SUI), a state portrayed by occasional loss of urine due to weakening of pelvic muscles, urinary sphincter or other factors such as childbirth and prostatectomy. SUI commonly manifests its symptoms during occasions that cause unexpected increases in intra-abdominal pressure such as laughter, sneezing or coughing. This remarkable finding has led to the rapid development of Duloxetine, wherein the year 2004, 2008 and 2010, the drug was approved by the FDA for diabetic peripheral neuropathic pain (DPNP), fibromyalgia and chronic musculoskeletal pain treatment, respectively [57,58]. As of the year 2011, Duloxetine has generated more than $9 billion in revenue.

### 4.4. New Hope for Orphan Disease Drug Development

Drug repositioning has paved the path for the development of orphan drugs for orphan diseases. Orphan diseases (ODs) are denoted as a disease with a rare occurrence, affecting only a small population, typically less than 200,000, in which no effective treatment is available [59,60,61]. This phenomenon is primarily due to the overall low drug demand [60,61], and the low availability of clinical trial subjects due to the rare occurrence [62]. Subsequently, orphan diseases have been overlooked by research and pharmaceutical industries as the investment of time and money may not be profitable [60]. The rapid increase of rare or orphan diseases estimated to affect up to 20% of the world’s population has driven governments and private pharmaceutical/biotechnology companies to explore cost-effective approaches by evaluating existing drugs [59]. To date, there are more than 7000 orphan diseases and the number is continuously expanding [63]. Drug repositioning plays a crucial part in this perturbing situation in exterminating life-threatening and debilitating rare/orphan diseases, pulling the gap closer between the availability of drug treatment to diseases within a population. 

To speed things up, a central medical research agency, the NIH, has been developed. The success and development of NIH are a result of an increased collaboration effort between multiple parties, such as academic institutions, governments and biopharmaceutical companies. To further assist the development of rare and orphan drugs, the NIH has created the NCGC Pharmaceutical Collection (NPC), in collaboration with NIH’s Therapeutics for Rare and Neglected Diseases (TRND) [60]. The prime mission of this collaboration is to assist the drug development body via the NPC Informatics Resource web browser, a comprehensive collection of more than 9000 drugs and compounds accompanied by over 200 drug target assays [60]. Besides that, to expedite drug repurposing for rare and orphan diseases, the U.S. Food and Drug Administration (FDA) has also established the Rare Diseases Repurposing Database (RDRD), by matching the FDA orphan database to the FDA drug and biological product approval lists. With these efforts, there were over 281 orphan drugs approved in 2014 [64]. With such a positive approach, possibilities for advance therapeutic in orphan diseases (ODs) can heighten to another success.

#### 4.4.1. Korlym’s Legacies on Orphan Disease

Cushing’s syndrome is an orphan disease that affects a minute population. The development of this disease occurs due to the prolonged exposure to glucocorticoids such as cortisol, which decimates the normal biological systems of the body. The most profound symptoms can range from excessive weight gain to dreadful acne, constant anxiety, hirsutism and insomnia [65]. The drug Korlym has successfully addressed this disease. Korlym is a repurposed drug approved in the 2012, where it was intended for abortion with the active ingredient, mifepristone which, is now considered a remedy for Cushing’s syndrome. In 2018, The Washington Post had interviewed a 40-year-old Georgia resident who suffered from Cushing’s syndrome. The drug Korlym helped this patient by blocking their body’s ability to process cortisol, thus relieving the disease’s symptoms. Korlym’s legacies have led to the development of an enhanced successor subtracting the side-effects, namely Relacorilant, a highly selective glucocorticoid receptor antagonist [66]. Relacorilant is now also in phase-I clinical trials for hormone-resistant prostate cancer [67]. 

#### 4.4.2. Everolimus on Rare Tuberous Sclerosis Complex

Most of the rare diseases correspond to orphan disease, but not always. One of the prime examples of the rare orphan disease is the Tuberous sclerosis complex [68]. It is a rare autosomal dominant genetic disease resulting from the mutations in the *TSC1* and *TSC2* genes, which encode for hamartin and tuberin, respectively [69]. This hamartin-tuberin complex regulates the mammalian target of Rapamycin (mTOR), which acts as a tumour-growth suppressor, controlling the cell growth and proliferation. It is highly associated with many non-cancerous tumours, known as hamartomas [70]. The prevalence of this genetic disorder is 1 in 6000 new-borns, where the genetic aberrant induces hyperactivity of the mammalian target of Rapamycin (mTOR) [71] and subsequently causes a spur of multiple hamartomas in organs such as the brain, kidneys, skin, lungs, eyes and heart [71]. These hamartomas can affect the function of many vital organs which result in symptoms including seizures, intellectual disability, developmental delay, behavioural problems, skin abnormalities, lung disease, kidney disease and even death. Everolimus is a drug that addresses this perturbing and fatal genetic disease. Everolimus was first approved in the 1960s to prevent solid organ transplant rejection, augment anticancer treatment regimens and to prevent neovascularisation of artificial cardiac stents. The FDA later approved the use of Everolimus in 2010, and 2012 for TSC-related subependymal giant cell astrocytoma and renal angiomyolipoma, respectively [72]. This drug successfully helped TSC patients by inhibiting the mTOR pathway, thus preventing the growth of the life-threatening hamartomas. Moreover, Everolimus has shown promising results in several clinical trials on the manifestation of TSC, such as facial angiofibroma, cardiac rhabdomyomas and lymphangioleiomyomatosis (LAM) [72].

## 5. Promising Novel Uses in Drug Repositioning

Drug repositioning can be done by a variety of approaches, such as different routes of administration, combining two different existing drugs for a new indication and enhancing or altering existing formulations [11]. For instance, a study conducted on a modified drug, carbamazepine ER (extended-release), has shown a fruitful outcome where there were significantly less patients who suffered from the adverse effects upon administration of Carbamazepine ER as compared to the traditional IR (immediate-release) at 6 versus 36 in 48 patients [6].

In some cases, drug repositioning captures a high return on investment potential and covers monetary loss in the initial unpropitious outcome of drug development. For example, the once-controversial Thalidomide that was initially intended for sedative purposes is now mainly used for specific cancer treatments. Together with its derivative Lenalidomide, manufactured by Celgene, it successfully captured a more than $2.8 billion global revenue. This translated to a superior return on investment as compared to traditional novel chemical entity/new molecular moiety (NCE/NME) drugs [73]. The NCE/NME drugs are the traditional drug discovery route where the discovered molecules would undergo clinical trials and await FDA approval for disease treatment.

The explored novel uses in drug repositioning has opened many possibilities on disease treatment, pulling the gap of treatment and diseases closer one-step to eradication. Also, it increases the quality of treatment, especially on highly mutated diseases, novel disease outbreaks and highly resistant and orphan diseases.

### 5.1. Novel Uses of Zidovudine for Viral Diseases

The development of a drug for highly mutated diseases such as Human Immunodeficiency Virus (HIV) has been an expensive and time-consuming process. To date, there are more than 30 drugs approved for HIV treatment. Each drug has a different approach, classified under six different classes, namely the nucleoside reverse transcriptase inhibitors (NRTIs), non–nucleoside reverse transcriptase inhibitors (NNRTIs), protease inhibitors (PIs), integrase inhibitors (INs), entry/fusion inhibitors and CCR5 receptor antagonists [74]. However, with the error-prone reverse transcriptase in HIV leads to increasing drug resistance, thus limiting drug efficacy. To make matters worse, drug resistance to HIV occurs more than one drug at a time as the resistance is based on the same mode of inhibition within the same drug classes, rendering the therapeutic potential ineffective [75]. This concomitantly increases the drug development costs and subsequently, the time expenditure [75]. In accordance with this situation, a fast, economical, yet effective drug developmental approach is prioritised. For instance, an anticancer drug, zidovudine/azidothymidine (AZT) was developed in the 1960s specifically targeting the Friend Leukaemia Virus (FV) that causes leukaemia has been successfully repositioned to be an antiretroviral drug for HIV treatment today [75,76]. It is grouped under the NRTIs and works by selectively inhibiting the reverse transcriptase found within FV and HIV, concomitantly inhibiting the production of cDNA from its RNA [75].

The new combination of AZT, together with other drug classes like the non-nucleoside reverse transcriptase inhibitor (NNRTI), Efavirenz (EFV), has reported promising results on multidrug-resistant HIV-1 patients [37]. To date, studies are continuously evaluating the potential of drugs to repurpose for HIV as it is often regarded as an incurable chronic disease [77,78]. For example, it was discovered that two current drugs, iodixanol, and sirolimus work synergistically at specific concentrations for HIV treatment via molecular docking simulations [78]. 

### 5.2. New Life to almost Extinct Auranofin

Drug repositioning is also important to eradicate multitudinous highly resistant diseases such as metronidazole-resistant Giardia Lamblia, which accommodated up to 20% of the treatment failure cases [68]. Giardiasis is an uncommon disease that affects millions of people, especially in developing countries where it can be transmitted via foodborne illness or person-to-person transmission [68]. Auranofin, an antirheumatic drug approved by the FDA in 1985 had declined in popularity in recent years mainly due to an improved formulation of newer antirheumatic drugs such as Methotrexate, Minocycline and so on. Also, the significant adverse effects over the long-term usage of Auranofin such as diarrhoea, vomiting, abdominal cramps and symptoms of hypersensitivity moved interest away from that particular drug. However, in a recent study, Auranofin exhibited antigiardial activity, especially on a metronidazole-resistant strain by blocking the giardia thioredoxin oxidoreductase, a crucial enzyme to counter oxidative damage in Giardia Lamblia [68]. 

Apart from that, Auranofin has also shown a positive outcome in fighting HIV. One of the stumbling blocks to eradicate HIV is the harbouring of memory CD4 T cells. For HIV to replicate, the virus must attach itself to the host’s CD4 T cells. Subsequently, it will then fuse with the CD4 T cells. The HIV will then take over the host cell’s translation machinery, incorporate its RNA into the cell and produce its viral proteins to assemble and release [79]. According to Lewis et al., Auranofin notably reduces the cell-associated viral DNA reservoir and induces cell death, especially on memory CD4 T cells in monkeys [79]. On a side note, a recent study evaluated the effects of Auranofin on melanogenesis [80]. This study assessed and discovered that Auranofin successfully inhibited key factors in melanogenesis by inhibiting tyrosinase activity, significantly down-regulating cAMP levels and increasing the number of melanosomes in immature stages. With this promising outcome, repositioning Auranofin can undoubtedly benefit patients by providing a cheaper and effective alternative drug against metronidazole-resistant giardiasis, HIV and hyperpigmentation. Table 1 summarises many such examples of revitalised drugs that were almost forgotten or were repositioned and gained a new life and purpose.

## 6. Future Prospects of Drug Repositioning for COVID-19 Pandemic

Considering drug repositioning to use as therapy for novel diseases is a huge advantage when the specific drugs are not yet available or developed. Among the significant challenges during the global fight against infectious diseases are the unpredictable emergence of disease and the lack of therapeutics for uncommon diseases. The most recent example is the novel coronavirus disease (COVID-19) which arose in 2019, affecting over 17,700,000 individuals worldwide [82]. During this time, medical practitioners are at a struggle to find a rapid cure for hospitalised patients to improve patient conditions and minimise outbreak severity. Drug repurposing has been highlighted as a more effective strategy for the search of a treatment for COVID-19 as it suits the urgency for the need of treatment [83]. 

COVID-19 is caused by severe acute respiratory syndrome Coronavirus 2 (SARS-CoV-2), a positive-strand single-stranded RNA virus with a genome size of approximately 29.8 to 29.9 kilobase pairs [84]. This virus’ genome is reported to have 14 open reading frames (ORFs) that encode for 27 viral proteins [85]. The most prominent viral protein in the structure of SARS-CoV-2 is the spike (S) protein or spike surface glycoprotein known for its large role in host cell membrane fusion and viral entry. Other SARS-CoV-2 structural proteins include the matrix protein (M), nucleocapsid protein (N) and the small envelope protein (E) [85]. Accessory proteins include (3a, 3b, p6, 7a, 7b, 8b, 9b and orf14). SARS-CoV-2, like many other viruses, is dependent on the host cell transcription and translation mechanisms for replication and production of viral proteins. 

Drug repositioning has played a large role in this case, and educated guesses were made to determine the best combination of existing antiviral therapies to treat the novel disease. In the early stages of the COVID-19 outbreak, doctors in Bangkok, Thailand, administered a combination of antiviral drugs Oseltamivir and Lopinavir/Ritonavir for Influenza and HIV to a Chinese woman who was infected with COVID-19, improving her condition shortly after [86]. Additionally, potential drugs such as the nucleoside analogue, Remdesivir peptide (EK1), RNA synthesis inhibitors (TDF, 3TC) and anti-inflammatory drugs have been suggested as potential drugs to be used in the treatment of COVID-19 patients in the time before a specific therapy is developed [87]. In addition, there have been many cases that followed which are now exploring the potential of antiviral drugs for other viral diseases to be used for COVID-19. Two HIV protease inhibitors, namely, Ritonavir and ASC09, are currently underway in clinical trials to treat COVID-19 [88]. Simultaneously, BrightGene Bio-Medical Technology, a Suzhou-based company, has announced that they will be manufacturing Remdesivir (GS-5734), an abandoned drug which was initially developed to treat Ebola virus, as an antiviral drug against COVID-19 [88]. 

Other drugs in consideration for COVID-19 treatment include Favipiravir and Ivermectin. Favipiravir, which was originally developed for influenza, when compared to Umifenovir (Arbidol), led to shorted latencies to relieve cough and pyrexia in COVID-19 patients [89]. Ivermectin, an FDA-approved broad anti-parasitic drug, has also been reported to have broad antiviral activity. An in vitro study, Caly et al. reported that a single treatment of Ivermectin expressed a significant reduction in SARS-CoV-2 viral load in a 48-h cell culture environment [90].

More advanced approaches using computational drug repositioning studies are currently underway to select already available antiviral drugs such as Carfilzomib, Eravacycline, Valrubicin and more to use as COVID-19 treatments [91]. This study describes the potential of the approved anticancer drug Carfilzomib for COVID-19 and other drugs such as antibiotics through studying protein docking sites, molecular dynamics simulations and binding free energies of approved drugs and drug candidates in clinical trials narrowing the drug search for COVID-19 [91]. 

In more severe cases of COVID-19, severe acute respiratory syndrome coronavirus 2 (SARS-CoV-2) develops. To combat this condition, additional drugs are required to be administered to an affected patient for recovery. Wu et al. performed an analysis of already available therapeutic targets that can be repurposed for SARS-CoV-2 treatment via systematically analysing the proteins encoded by SARS-CoV-2 genes [92]. In this study, 78 antiviral drugs were analysed against target ligands to reveal the best likely candidates of drugs suitable for potential usage against SARS-CoV-2. 

Very recently, a study by Elfiky has used an up-to-date molecular docking strategy to elucidate the drugs Ribavirin, Remdisivir, Sofosbuvir, Galudesivir and Tenofovir as those that are potent against SARS-CoV-2 as they were predicted to bind to the RNA-dependent RNA polymerase (RbRp) of the newly emerged coronavirus [93]. All the aforementioned drugs are possible inhibitors of RbRp and may result in the reduction of infection severity. Multiple software were used to validate the RdRp model. These include the PROCHECK, Verify 3D, PROVE, ERRAT and others [93]. Upon validation of the optimised RdRp model, the AutoDock Vina software was then utilised for the docking experiments. The methods and results of this work have been elaborately explained in their research article. 

### Repurposing the Antimalarial Drug Chloroquine for SARS-CoV-2/COVID-19 Treatment

Recently, there have been discussions on repurposing the antimalarialdrug Chloroquine (CQ) or its alternatives CQ phosphate or Hydroxychloroquine (HCQ) for the treatment of SARS-CoV-2 patients. Initially, CQ and HCQ were considered for their therapeutic potential due to previous reports on their strong anti-SARS-CoV activity in vitro after the 2003 coronavirus outbreak [94]. Other derivatives of CQ, such as HCQ sulphate have been reported to reduce inflammation in autoimmune diseases [95]. Such information ignited interest in the potential of CQ and HCQ to treat COVID-19 ill patients. In this section, we describe the general mechanism of action of CQ against malaria, which was the original disease it was produced for. In addition, current research and clinical trials that have been conducted for repurposing CQ or HCQ for COVID-19 are highlighted. 

The exact mechanism of action for CQ and HCQ are yet to be fully discovered, especially in the context of this viral outbreak. The classical mechanism of action for CQ as an antimalarial drug involves preventing the polymerisation of Heme residues [96]. *Plasmodium falciparum* is a parasite that causes malaria disease in humans. For this parasite to grow whilst living within a host, it depends on amino acids from haemoglobin for essential nutrients. Once haemoglobin is broken down into amino acids, the Heme residues remain, which are toxic to the parasite. To protect itself from the toxic effect of Heme accumulation, the parasite polymerises Heme into Hemozoin. Therefore, CQ interferes here to prevent this polymerisation, thus leading to the accumulation of Heme within the parasite, consequently killing the parasite [96,97]. The mechanism of action of CQ against other parasites may be similar or entirely different, and therefore, further studies to elucidate the mechanism of action against other parasites such as viruses are always required. CQ has been reported previously to block virus infection in a few ways such as through increasing the pH in endosomal environments while virus-cell fusion occurs or by interfering with SARS-CoV surface receptors via glycosylation [98]. Such studies provide an explanation on the role of CQ in a viral life cycle, which supports its candidature potential to be repositioned for a COVID-19 treatment.

A commentary by Lamballerie and Touret summarised the studies conducted thus far on CQ and its antiviral potential in the past suggesting that it has little or no effect on many acute viral diseases such as influenza, dengue, Ebola, HIV and Nipah viruses. However, in their discussion, it was highlighted that in the case of Hepatitis C infection, HCQ used in combination with pegylated interferon and ribavirin resulted in a positive effect on reducing the viral load [99]. 

In vitro studies are now being performed to evaluate the effects of CQ or HCQ on SAR-CoV-2. One study has concluded that HCQ was found to have more potent antiviral activity as compared to CQ alone [100]. A recent in vitro study has tested the effects of five already available drugs against 2019-nCoV infection, namely ribavirin, penciclovir, nitazoxanide, nafamostat and CQ [91]. The highlight of their results has found that two of the drugs, ribavirin, and CQ showed excellent potential in blocking virus infection at low drug micromolar concentrations. Another study recommends the usage of CQ phosphate as the potential treatment of COVID-19 in this time as it has exhibited efficiency in treating COVID-19 associated pneumonia presented in patients. In addition, CQ has been considered the potential drug of choice as it can be produced on a larger scale for global distribution compared to other potential drugs against COVID-19 such as Remdesivir [87]. Moreover, it has a proven safety record for patient usage and is relatively low cost as compared to the other listed drugs. HCQ sulphate has been studied for its antiviral effect on SARS-CoV-2 infection and has been compared with CQ alone for cytotoxicity in vitro using African green monkey kidney VeroE6 cells [87]. This study has successfully shown that HCQ sulphate is effective in inhibiting SARS-CoV-2 infection and may be a better option for consideration in clinical trials for patient administration as it has a less toxic effect on cells as compared to CQ alone [87]. In contrast to this result, another study evaluated HCQ sulphate for SARS-CoV-2 antiviral effects and concluded that although overall clinical scores did decrease in COVID-19 positive ferrets after HCQ treatment, little effects were observed in virus titres of nasal washes, stool specimens and respiratory tissues when compared to other treatments or phosphate-buffered saline (PBS) treated groups [95]. In addition to using HCQ, a study by Gautret et al. evaluated the combination of HCQ in combination with the antibiotic azithromycin. This study has reported that the combination of these two drugs is associated with more efficient viral load reduction and disappearance of COVID-19 in twenty patients [14]. Despite the many studies mentioned here, it is yet uncertain whether CQ or HCQ are indeed completely effective in treating COVID-19 as some trials and studies have yet to conclude the certain effects. As with most drugs in consideration for repositioning, thorough evaluations may take some time in order to declare them safe and effective for treatment. A highly regarded concern that comes along with drug selection are the side-effects or adversities of the drug to be used for treatment. Table 2 summarises the side-effects along with other information for some of the current medications under the drug repositioning spotlight for COVID-19. The side-effects mentioned are specific to observation from COVID-19 patients in each clinical trial or study.

## 7. Future Prospects of Drug Repositioning

### 7.1. Path to Drug Personalisation

Human diseases are expressed by underlying complex mechanisms that can be developed or obtained from many sources such as aberrations in genetics, infectious diseases, degenerative disease and more. Diseases often involve many complex cellular pathway cascades, which can vary in certain individuals. In diverse human populations, each individual has a unique set of inherited or non-inherited genetic abnormalities that could result in certain individuals to respond less or not at all to general treatments or drugs. Approved drugs can be invalid for a specific individual if they are deficient in a particular drug target and may not respond to the common way to a particular drug. This situation entails the requirement of personalised medicine to tailor for each patient, as drug efficacy can defer substantially in different gene profiles due to heterogeneity in human disease [109]. To reduce the lack of drug efficacy, drug repositioning plays an important role. Together with the advancement of next-generation sequencing technologies, personalised genomic studies can be carried out with an affirmative approach. This can result in the best medicine accompanied by high efficacy at the lowest toxicity levels possible to the specific individual in contrast to conventional treatment that aims for maximum tolerated doses. For instance, non-small cell lung cancer (NSCLC) accounts for 85–90% of all lung cancers [110]. In 2007, it was first reported that there is a fusion gene in NSCLC, which comprises portions of echinoderm microtubule-associated protein-like 4 genes (*EML4*) and anaplastic lymphoma kinase gene (*ALK*) in ∼7% of patients with NSCLC. This was different from the common NSCLC with epidermal growth factor receptor mutation. Crizotinib, a small-molecule kinase inhibitor, which was intended to treat ALK-positive anaplastic large-cell lymphoma, is now repositioned to treat this small subset of NSCLC [81]. Without the advance screening of biomarkers in each NSCLC individual, this small subset would not be successfully discovered, and the common EGFR tyrosine kinase inhibitors would have been prescribed to all NSCLC patients albeit the limited efficacy in patients with *EML4-ALK* alteration. Recently, the drug Crizotinib (Pfizer’s Xalkori) successfully obtained FDA approval as a treatment for patients with NSCLC with *MET* exon 14 skipping which occurs in approximately 3% of NSCLC tumours and *ROS 1*/anaplastic lymphoma kinase-positive resistant systemic anaplastic large cell lymphoma (*ROS-1/ALK*-ALCL) [111]. Hence, repositioning the drug based on stratification is important in accordance with the appropriate biomarkers for each individual.

### 7.2. Challenges of Drug Repositioning

To no surprise, drug repositioning has its challenges despite being an advantage over traditional de novo drug discovery. Drug patents can play a critical role in drug repositioning. “Composition of matter” is a compound patent protection, which protects the rights of whoever invents or discovers any new processes, machines or new compounds but not limited to new formulations, recipes or materials for 20 years [112]. Hence, patenting in drug repositioning can be arduous, especially when the original indications have already been trademarked ahead of new competitors [113]. There are many ways to approach the existing COM patent with drug repositioning. For instance, the company Zalicus in collaboration with Sanofi Aventis Paris developed Prednisporin, which combines two old drugs, glucocorticoid Prednisolone acetate and the immunosuppressant Cyclosporine A for the treatment of allergic conjunctivitis [63]. 

Apart from this, the lack of participation and an insufficient amount of pronounced repositioning projects, especially from big renowned pharmaceutical companies contribute a significant challenge in drug repositioning. This scenario is due to the lack of faith in prospective projects, the likelihood of failure and huge time and money investment in these projects. Interestingly, the role of drug repositioning was mostly initiated by companies other than the primary inventor [110]. For example, Thalidomide, a complete failure in drug development history was brought back to life by the company Celgene to treat Multiple Myeloma Nodosum Leprosum and Erythema with the exclusion of pregnant women, but not by the inventor company Grünenthal [73,112,114].

To outline and implement clinical trials pilot projects, a considerable amount of funding and expertise especially on unique medical conditions are always important abut can be difficult to obtain for specific projects required by biotechnology and pharmaceutical industries. Nonetheless, out-licensing may address this hurdle contingent upon the eagerness of small biotech companies or research organisations to take up the aforementioned risk, which, to date, is limited [112]. 

Besides that, it is especially difficult to obtain funds when the candidate drug to be repositioned is one that has been rejected for other reasons in the past, rendering faith in the outcome weaker. Unfortunately, even though most countries are enthusiastic about research breakthroughs in healthcare, not many countries have excessive funding to put aside for such projects. For instance, there are sponsored trials for drug repositioning in the U.S. by the National Institutes of Health (NIH); however, the same advantage may not be available in European or Asian countries. 

Despite the fact that there are numerous advantages to drug repositioning over the traditional de novo approach, it is not all successful. There have been a few instances where drug repositioning projects have failed. A kinase inhibitor, Bevacizumab, which was, developed to treat gastric cancer, failed to show efficacy in phase III trials despite being well studied and repositioned to several other types of cancers [115]. 

Although large datasets are available for early stages in identifying drug targets, data access and integration have been acknowledged as an issue in the pipeline as better integrative platforms are needed for data analysis [11]. Additionally, a lack in reporting of data or patients from the clinical settings may pose a limitation in identifying drug targets for rare or orphan diseases, making the practice of drug repositioning much harder. On a related note, as some drug targets are identified via in vitro experimentation, another limitation includes failure in pursuing a potential candidate drug that goes beyond the initial studies [11]. 

The drawbacks, ranging from the notorious perception of drug repositioning, funding requirements, demand on considerable amounts of expertise, unexpected side-effects, shortages of potential drug candidates to the lack of integrative platforms for data analysis, has put drug repositioning into a difficult state. However, with the close collaboration between the pharmaceutical companies, governments and scholars, together with the advancement of computational methods today, the drawbacks of drug repositioning can be positively mitigated.

## 8. Conclusions

Despite the drawbacks and stigmas, drug repositioning still holds significant interest and potential for pharmaceutical companies and researchers for new discoveries and a new life for old drugs. New high throughput screening techniques coupled with advanced computational methods such as signature matching, molecular docking, GWAS and pathway mapping, old drugs should be relooked at and reconsidered with the aim of repositioning. Drug repositioning brings many benefits ranging from high capital returns, low developmental cost and a relatively low risk of failure to significantly reduced time to reach industrial-scale production and commercialisation. Besides, drug repositioning opens new opportunities for orphan diseases, pandemic outbreaks and highly resistant diseases. This approach is especially evident in repositioning drug Thalidomide, where a safe development resulted in new hope for multiple myeloma and brought in revenue of $271 million USD. Also, drugs with modified formulations such as Valproate and Concerta, for instance, attained enhanced activity as compared to their initial development for epilepsy and hyperactive disorder, respectively. Orphan diseases such as Cushing’s syndrome and TSC obtained promising treatments through the repositioned drugs Korlym and Everolimus, successfully alleviating the symptoms in the affected patient. Apart from that, drug repositioning allows the development of personalised medication, which benefits not only the biotechnology and pharmacological companies but also the affected patients, who are provided with a more affordable drug with an increased efficacy against their aliment. 

It is essential to highlight that drug repositioning attempts are crucial for the search for effective therapy in an urgent pandemic such as the COVID-19. Research and clinical evaluations are strongly urged to continue to evaluate the repositioning potential of existing drugs which yield significant alleviation in disease symptoms. In the current quest for a cure, a significant consideration is being given to existing antiviral drugs such as Ribavirin, Favipiravir and Remdesivir, amongst many other drugs. A call for more government participation in sponsoring research and development projects, via research grants, tax incentives, infrastructure or clinical cooperation support can bring exponential growth to future projects in drug repositioning.

## Figures and Tables

**Table 1:** Summary table of repositioned drugs and their new roles.

Drug	Original Medication	Repositioned Medication	References
Auranofin	Rheumatoid arthritis	Metronidazole resistant Giardiasis	[68]
Crizotinib	Anaplastic large-cell lymphoma	Non-small-cell lung cancer (NSCLC)	[81]
Duloxetine	Major depressive disorder	Stress Urinary Incontinence (SUI)	[5]
Everolimus	Prevent solid organ transplant rejection, Augment anticancer treatment regimens, Prevent neovascularisation of artificial cardiac stents	Tuberous sclerosis complex [22]	[72]
Korlym	Abortion	Cushing’s Syndrome	[66]
Minoxidil	Hypertension	Androgenic alopecia (Hair loss)	[51]
Sildenafil	Angina, chest pain/discomfort	Erectile Dysfunction	[55]
Thalidomide	Morning sickness pregnant women	Multiple myelomas	[13]
Zidovudine	Anticancer agent	Antiretroviral agent-HIV	[75]

**Table 2:** Summary of popular drugs under consideration for repositioning for COVID-19.

Drug	Initial Purpose	Side-Effects in COVID-19 Patients	Potential Therapeutic Mode of Action for SARS-CoV-2	Clinical Trials for COVID-19	Reference
**Chloroquine/Hydroxychloroquine**	Antimalarial medication	Side-effects may arise with toxic dosage for patients with cardiovascular disorders Excessive prolong in QTc intervals which leads to ventricular arrhythmias Gastrointestinal upset Retinal toxicity and myopathy	Impairs replication of virus by interfering with endosome-mediated viral entry or other pH-dependent viral replication steps	Currently ongoing	[101,102]
**Favipiravir**	Broad-spectrum inhibitor of viral RNA, i.e., Influenza	Raises serum uric acid Diarrhoea	Inhibits viral RbRp in the genome replication process	currently ongoing	[23,89,103]
**Ivermectin**	Broad-spectrum anti-parasitic agent	Not reported for COVID-19. Predicted as: Nausea, rashes, dizziness Fever and tachycardia	Nuclear transport inhibitory activity of viral proteins	In vitro studies	[90,104]
**Remdesivir**	Antiviral activity against RNA viruses, i.e., Ebola virus	Abnormal liver function Renal impairment Hypotension, diarrhoea and rashes	Acts as RbRp inhibitor to target viral genome replication process	Currently ongoing	[105,106,107]
**Baricitinib**	An orally bioavailable inhibitor of Janus kinases 1 and 2 (JAK1/2), with potential anti-inflammatory, immunomodulating and antineoplastic activities. Typically used to treat autoimmune disorders such as rheumatoid arthritis.	Not reported for COVID-19. Previous original indication. Nausea Blurred vision Cold sores Shingles Possible increase in upper respiratory tract infection Hypercholesterolemia Hypertension Urinary tract infection	A high-affinity NAK inhibitor, especially on AP2-associated protein kinase 1 (AAK1), a pivotal regulator of clathrin-mediated endocytosis involved in viral entry at the lungs	Currently ongoing	[39,108]
